# Disdainful Hookups: a Powerful Social Determinant of Health

**DOI:** 10.1007/s11524-023-00765-4

**Published:** 2023-08-03

**Authors:** Lidia Puigvert, Sandra Racionero-Plaza, Garazi Lopez de Aguileta, Itxaso Tellado, Silvia Molina, Miguel Ángel Pulido-Rodríguez, Leire Ugalde, Ramon Flecha

**Affiliations:** 1 Department of Sociology, University of Barcelona, Barcelona, Spain; 2 Institute of Criminology, University of Cambridge, Cambridge, UK; 3 Department of Pedagogy, University of Vic-Central University of Catalonia, Vic, Spain; 4 Department of Curriculum & Instruction, University of Wisconsin-Madison, Madison, WI USA; 5 Department of Pedagogy, Rovira I Virgili University, Tarragona, Spain; 6 Department of Humanities and Pastoral Care, Ramon Llull University, Barcelona, Spain; 7 Department of Didactics and School Organization, University of the Basque Country, Donostia-San Sebastián, Spain

**Keywords:** Peer group, Coercive dominant discourse, Gender violence, Adolescence, Peer harassment, Violent sporadic relationships

## Abstract

The health consequences of gender violence, a global health and social problem, are increasingly studied. Among its roots, research has identified a coercive dominant discourse imposing the idea that masculinities and relationships marked by abuse and domination are more attractive than egalitarian ones. To prevent the health consequences of gender violence, it is necessary to understand the factors that lead many adolescents to fall into it. This study aims to identify the specific mechanisms by which the coercive dominant discourse manifests in the peer group and its consequences for adolescents. Forty-one 15- and 16-year-old female adolescents from three high schools in Barcelona participated in the study. Eight communicative discussion groups were conducted to deepen on participants’ perceptions regarding how peer interactions promote the learning of attraction to violence in sexual-affective relationships. The results show that the participants perceived and experienced different types of coercion to have violent relationships in their peer group interactions. Those interactions fostered the reproduction of the association between sexual-affective attraction and males with aggressive attitudes and behaviors. Many peers coerce others to have disdainful hookups which have very negative health consequences for the victims, including suicidal ideation and committing suicide. Some peer groups become a risk developmental context for female adolescents as far as they foster the coercive dominant discourse, push some young women to engage in violent sporadic relationships, and even harass some others afterwards. This clarifies the importance of peer group-level interventions when addressing the health consequences of gender violence in adolescence.

## Introduction

The scientific literature has found multiple relations between gender violence and several health consequences [1, 2], including chronic pain, increased risk of sexually transmitted diseases, depression, and even suicidal tendencies, among others. Given the graveness of this public health problem, the prevention of such health outcomes should include the prevention of violence against women and girls. A key for preventing health consequences related to gender and sexual violence is to analyze the factors that lead adolescents to subdue to it. This article presents for the first time the analysis of one of the specific mechanisms that promote such violence: peer pressure. Identifying and eliminating peer pressure is a necessary step in the prevention of the health outcomes related to gender violence.

Although it is hard to determine the exact prevalence of gender and sexual violence, it is estimated that around 27% of women aged 15 to 49 worldwide have suffered physical or sexual violence [3, 4]. Considered a global public health issue [5], research has reported several physical and mental health consequences of suffering such violence. Among others, chronic pain [6]; gastrointestinal disorders [2]; self-harm and suicidal ideation or attempt [5, 7]; substance abuse [8]; an increased risk of suffering HIV [9]; depression [10], or psychological distress [11] have been found as consequences of suffering gender and sexual violence. These data are even more alarming as violent sexual-affective relationships have a strong prevalence among adolescents and youth [4].

Among other causes [12], research has identified a coercive dominant discourse which is socially constructed and presents men with violent and disdainful attitudes and behaviors as more attractive and sexually desirable than those with egalitarian ones [13, 14]. This discourse is learned via socialization, as it is present in central developmental contexts in childhood and adolescence [15]: peer interactions, the media, social media, magazines, and literature for young people [16, 17]. Research in various fields, including socioneuroscience, has shown that continued exposure to this model makes some girls build a pattern of attraction towards violent boys and embark on unhealthy sexual-affective relationships [18–20]. Importantly, even if this model can be identified in stable and sporadic relationships (often referred to as hookups), research has shown a greater tendency to choose violent boys for hookups and non-violent boys for stable relationships [21].

The peer group plays an essential role in girls’ and boys’ socio-emotional development [22]. Social interactions in the peer group can become one more context of socialization into the coercive dominant discourse [23, 24]. Research has shown that when adolescents talk with interest and desire about boys with violent attitudes and behaviors, their attraction patterns are reinforced to be submissive to the coercive dominant discourse [25], increasing the risk of engaging in violent and disdainful hookups.

Still, little has been examined regarding specific interactions in the peer group that foster preferences for sexual-affective relationships with boys with violent attitudes and behaviors as well as promote engagement in such type of disdainful hookups. Given the health consequences of such violence among adolescent females, it is urgent to examine in depth the mechanisms by which peer group pressure and coercion occurs. The research reported in this article addresses this gap by aiming at describing coercive interactions and dialogues among a group of 15- and 16-year-old adolescents that foster disdainful hookups as a required step to prevent health consequences related to gender violence.

## Methods

### Participants

The study sample consists of 41 female students from three different high schools in Barcelona (Spain), two public and one semi-private. The participants have diverse cultural and ethnic backgrounds, and all attended 4th grade (ages 15 and 16) at the time of the study.

The inclusion criteria were (a) students had provided informed written assent and (b) their parents had provided written informed consent for participating in the project. Participants in the communicative discussion groups were randomly selected. All the schools decided to participate freely without any economic incentive.

### Instruments

Eight communicative discussion groups were carried out. The communicative discussion group allows a collective interpretation of reality through egalitarian dialogue among all members. For carrying it out, a natural group of people who have some common link is created in a natural environment of trust. In our case, all participants belonged to the same class, and the discussion groups were conducted in their high schools. The researcher was integrated into the group and helped to facilitate dialogue between peers, ensuring that communication was not subject to the imposition of some opinions on others.

### Material and Procedure

Before implementing the study, ethics committee authorization for the study protocol was obtained. Once the protocol was approved, information sessions were held in the high schools with the directors, teachers, parents, legal guardians and the adolescents themselves. In these sessions, as well as during the whole project, researchers gave all the necessary explanations and answered any questions. Afterwards, the informed consent was completed. These consents ensured voluntary participation in the study, the anonymity and privacy of the participants, and the possibility of withdrawing at any time. To ensure anonymity, the use of codes and pseudonyms was guaranteed. The consent form explained the details of the study. The directors of the center also signed a letter that included all this information.

The project design and implementation process were based on the communicative methodology, which has been shown to achieve social impact in gender violence research [26, 27]. This methodology implies the participation and egalitarian dialogue among those involved in the research.

### Data Analysis

The following categories compound the coding scheme*: (1) Harassment in adolescent peer groups to engage in sporadic and violent sexual relationships*; (2) *Harassment to destroy stable relationships.* Within these two main categories, subcategories have been established: (1a) *Inciting to reproduce violent attitudes in girls*: attitudes which encourage girls to imitate the behavior of violent boys in sexual-affective relationships in which they hook up with different people and then talk badly about them; (1b) *Harassment leads them to hook up with violent boys*: friends pressure girls to hook up with violent boys, presenting them as attractive; (1c): *Pressure to link attractiveness with violent males and to normalize violent attitudes*: friends coerce girls to end up seeing violent boys as attractive and normalize their violent attitudes; (2a): *Strategies for harassing friends with stable relationships*: friends coerce girls who had a healthy relationship to hook up with violent boys; (2b): *Process and consequences of bullying to break stable relationships*: strategies used to harass people to hook up with violent boys and the consequences that it entails. A discourse analysis of the specific interventions that respond to the coding schemes has been carried out.

### Ethical Approval

The study was conducted following the guidelines of the Declaration of Helsinki (World Medical Association, 2013) and Horizon 2020 (European Commission). The study protocol was revised and approved by the Andalusia Government, specifically by the Research Ethics Committee of the Virgen de la Macarena and Virgen del Rocío Hospitals.

## Results

### Harassment in Adolescent Peer Groups to Engage in Violent Sporadic Sexual Relationships

#### Inciting to Reproduce Violent Attitudes in Girls

A clear example that shows this is when we found girls who incite the imitation of violent boys’ behaviors among their female friends. Under the argument that “if boys treat girls badly, they should do the same,” some girls consider that the best way to confront the male model of abuse is to pressure other girls to reproduce it instead of rejecting it, expanding its damage. In the following quote, the researcher asked some participants in what ways they usually help their friends avoid toxic relationships and the bad consequences derived from them. Answering to the researcher, Sara says that she would encourage her friends to hook up with different boys and then talk badly about them:

Researcher: If, for example, you see that she [a friend] is going to hook up with a guy like that [with violent behaviors], and then they are going to talk about her so badly and say of her that kind of things, how do you help her?

Sara: Well, I would also tell them that if guys can do that with girls, why can't girls do that with guys, you know? If guys can make out with anyone and they can talk bad about her, then girls can do the same.

#### Peer Coercion Can Lead to Disdainful Hookups

Participants confirm the great influence that friends’ advice and opinions can have on the behavior of some young people, explaining that friends’ pressure can be so influential that some girls end up hooking up with a violent boy when they really did not want to. In this sense, Ana refers to the influence of “false friends” who encourage girls to hook up with boys who will not treat them well:

Researcher: Do you think that maybe girls who, as you said, don’t like those [violent] guys, who really don’t want to have a hook up with them, might ending up having them because of pressures from their group of friends?

Several girls: Yes

Ana: From fake friends.

Along the same lines, Nerea describes that her own “friends,” even knowing that the girl has no interest in a boy, insist on getting her to hook up with the toxic popular guy by telling her that this boy is “handsome.” Nerea notes that her friends want the girl to “fall into” that kind of disdainful hookup and do what she would not do without that peer pressure.

Nerea: Well, the boy wants [to have sex]. (…) So, we insist to that girl like “come on, that boy wants to have sex with you, he is handsome, that boy has a certain level, that boy hangs out with so and so”, until the girl falls for it and then it happens.

Nerea employs the first person of plural, “we,” indicating that she herself has been engaged in such coercive behavior.

#### Pressure to Link Attractiveness with Violent Males and to Normalize Violent Attitudes

Another type of pressure that participants identify in their interactions is making a girl like a boy with violent attitudes and behaviors that she does not like. The constant repetition to a girl saying “who she really likes” can make her end up liking him, according to the participants. This type of coercion can cause a girl to learn attraction towards an aggressive boy for whom she previously felt nothing. In this way, in addition to generating a link between attraction and violence that may affect future choices, the peer group pushes girls into a toxic relationship in which they can be seriously damaged.

Sonia: It’s like when they tell you that you like this guy, you like this guy, when they say it all day long, you end up believing that you like this guy, but it’s not true.

Participants also shared that these types of pressure can lead to normalize and accept violent behaviors and confuse them with love, suggesting that abusive behaviors are due to love. As we can see in the following quote, the group of friends can generate a discourse of acceptance and normalization of violence that is very dangerous.

Alicia: Well, for example, you are dating the popular guy and he is mistreating you, and you go to your friends and say “he doesn’t treat me well and this and that”, and they tell you, “you are crazy, he is very nice, he is very kind”.(…) and then you think “well, he does it because he loves me” and there you have the problem.

### Coercing and Harassing Female Friends with Stable Relationships to Have Disdainful Hookups

#### Tagging the Girl with a Stable Relationship as Boring and Instigating Fear of Losing Friends

The strategies reported by participants refer to making girls feel that being in a positive stable relationship is “boring.” Some girls suffer peer pressure to cheat on their boyfriends under the premise that “fun” is in having hookups with boys with violent attitudes and behaviors. Lucia described how her friends tagged her as “boring” for spending time with her boyfriend: “They tell you ‘you are getting very boring, you spend too much time with him’.”

Eva explained that this pressure is so high that the girl suffering it, out of fear of losing her friends, might end up hooking up with someone she does not want to do it with and cheating on someone she loves.

Eva: I have seen many cases. I have seen friends who give you bad advice. So while you were with him [boyfriend], the others would tell you “no, we are going out” and all that, and they would take you to the bars or “come to the bar, you can be with other guys there, I mean, with others, your boyfriend is not there”. And you’re there, they take you there, and when you’ve had too many drinks, they make you hook up with that guy. And you do it, so you don’t lose your friends.

Eva says that the friends “take the girl” to the bar and “make her hook up with this boy,” illustrating it is something the female friends force this girl to do but not something that she decides to do freely. On the other hand, the girl victim of this coercion is aware of the racketeering that exists behind these acts, which is evidenced when Eva says that the girl does it to “not lose her friends.”

#### Going Further: Taking and Disseminating Pictures Without Consent

Some participants reported that those same friends who have pushed the girl up to that point can take pictures of the girl; those can be photographs of the girl and the boy intimating or they can be of them talking or next to each other, not hooking up, but it could seem that way.

Amaia: They take pictures of you or they tell it to everyone.

Silvia: Or they post it.

Rosa: Maybe you’re not even hooking up with him, but they [the pictures] catch you in a position that…

Silvia: Yes, that it seems that you’re kissing him…

Maria: Or that you’re very close to him and it looks like something that it is not…

Participants reported that those same friends send the picture, first to the girl’s boyfriend and then upload it to the Internet or send it to other people, people who know the girl and her boyfriend, making it public for everyone. This behavior gives rise to more violent episodes between people, involving the girl’s boyfriend:

Rosa: They send [the picture] to the boyfriend.

Eva: They upload it to the internet; it gets to the boyfriend. And the boyfriend wants to fight with the other one or they can end up beating each other up. Some people are very aggressive.

On the one hand, this narrative shows that peers try to damage the girl’s stable relationship by sending the photo to the boyfriend. However, bullies are not satisfied with causing damage to the couple’s relationship, but rather amplify the impact of the damage caused by uploading the photo to the Internet. Once this is done, this photo will remain in the networks forever, leaving an indelible mark of the event in the history of that girl and of her future family. As participants explained, Internet allows anyone to “share, save or capture” the photograph. This makes the victim perceive the harassment as endless. This is what Eva and Maria also shared:

Eva: Well, that photo will be there all your life, it will be eating you up until you die. The picture will follow you to your grave.

Maria: It has been shared, it has been saved, screenshots have been taken…

Eva: There are people who end up committing suicide because it hurts so much.

Eva warns of how the photo, used as a tool to damage the girl, will always be within the reach of whoever wants to use it until “the grave.” Eva is also aware of the psychic damage caused by this event, saying that “it will be eating up you until you die.” The psychic damage that this kind of events generate would be so unbearable that, as Eva says, “there are people who end up committing suicide because it hurts a lot.”

## Discussion

This study sheds light on the role that power interactions among adolescents in peer groups can have in learning the coercive dominant discourse and engaging in abusive sexual relationships. Such peer pressure is one of the mechanisms that promotes gender and sexual violence; it is therefore necessary to address it to prevent and eliminate health outcomes related to violence against women and girls.

Our findings suggest that some girls influence their friends to reproduce the model of toxic masculinity [28]. The attitude of violent boys who make out with girls and then talk badly about them is criticized by the study participants. However, they believe that this attitude is rewarded in boys, often labeling them as “popular” in the peer group. On the contrary, those men’s environment criticizes girls who act that same way [15]. Far from trying to transform the abusive behavior that occurs in these relationships, our participants shared that some girls choose to encourage their female friends to act in the same way.

The fact that the peer group speaks well of boys who represent a violent masculinity is of great importance. Several participants reported feeling pressured to like and even hook up with boys they did not like first. They mention two types of strategies for this purpose. One is to constantly repeat to a girl that she likes a guy until she ends up questioning her own feelings, saying yes to respond to the pressure, or believing that she really likes that guy. A second strategy is to praise those boys by showing that they are valued in the peer group, saying things like “he’s got standards” or “he’s cute.” This type of language used among the peer group to talk about those boys responds to what research has called “language of desire” [15, 26]. The “language of desire” has been shown to have greater impact on adolescents’ affection and behaviors than the “language of ethics.” The former predominates in the dialogues in the peer group, in the media, etc. However, the “language of ethics” [29], used in contexts such as the family and the school, speaks of sexual-affective relationships from an ethical perspective in which what is “convenient” is described, often as boring and moral [21, 29].

Pushing girls to have disdainful hookups means highly raising their likelihood to be victimized from gender and sexual violence. Symptoms such as chronic pain and gastrointestinal symptoms [2, 6], depression and anxiety [10], suicidal ideation [7], or cognitive and emotional damage [30] are only some of the negative health outcomes related to gender violence. Our study also advances this knowledge as it shows that, according to our participants, peer pressure to have disdainful hookups can imply a series of strategies that may include bullying, such as taking photos of the disdainful hookup without consent and sending them to the girl’s boyfriend and to her social context. One of the consequences of such bullying, as reported by our participants, is the victim’s suicidal ideation and committing suicide. Our study advances that peer group pressure to have disdainful hookups can relate to severe psychological distress and suicidal ideation.

A final type of coercion detected in the data was the pressure against girls in stable relationships to break them. Stable relationships are presented as “boring” by many adolescents, pressuring girls who have them to go out and have disdainful hookups. Under the premise that “fun” is making out with guys with disdainful and violent attitudes, we have been able to collect examples of how some girls are pushed to have disdainful hookups with them.

The present study has limitations. One is that although previous research on the coercive dominant discourse has shown its existence in different countries and contexts, the research reported here is a qualitative study and its findings cannot be generalized. Future research can address this limitation by inquiring into this topic in different countries and with adolescents with other ethnic and racial backgrounds. Moreover, further research should examine protective peer interactions that prevent adolescents from engaging in disdainful hookups and thus protect female adolescents from the health consequences of suffering gender violence.

Despite the limitations, the present study shows evidence on the ways in which peer pressure can lead many adolescent and young girls to have disdainful hookups where they suffer multiple forms of violence. In this way, this study sheds light on peer pressure an essential element that researchers, policymakers, educators, and different social agents and citizens need to take into account and address for the prevention of the health consequences of gender and sexual violence.

## Data Availability

Anonymized data will be available in Zenodo open access database.
